# A Unique Homo-Hexameric Structure of 2-Aminomuconate Deaminase in the Bacterium *Pseudomonas species AP–3*

**DOI:** 10.3389/fmicb.2019.02079

**Published:** 2019-09-06

**Authors:** Yanjuan Chen, Yiping Chen, Hua Jiang, Deren Lu, Tingting Hu, Gang Bi, Yuping Ran, Baofeng Yu, Hui Dong, Dan Su

**Affiliations:** ^1^State Key Laboratory of Biotherapy and Cancer Center, West China Hospital, Sichuan University and Collaborative Innovation Center of Biotherapy, Chengdu, China; ^2^Department of Dermatovenereology, West China Hospital, Sichuan University, Chengdu, China; ^3^Department of Biochemistry and Molecular Biology, Basic Medical College, Shanxi Medical University, Taiyuan, China; ^4^Key Laboratory of Tianjin Radiation and Molecular Nuclear Medicine, Institute of Radiation Medicine, Chinese Academy of Medical Sciences and Peking Union Medical College, Tianjin, China

**Keywords:** homo-hexamer, 2-aminomuconate deaminase, YjgF/YER057c/UK114 family, RidA subfamily, *Pseudomonas species AP–3*

## Abstract

The bacterium *Pseudomonas species* sp. *AP–3* is one of several microorganisms that are capable of using 2-aminophenol as its sole source of carbon, nitrogen and energy. Several 2-aminophenol-metabolizing enzymes have pivotal roles in the biodegradation of aniline and its derivatives as environmental pollutants in *Pseudomonas*. The bacterium *Pseudomonas* sp. *AP–3* recruits a unique 2-aminomuconate deaminase (AmnE) to hydrolyze 2-aminomuconate to 4-oxalocrotonate, and releases ammonia in the modified meta-cleavage pathway by forming various compounds—including acetaldehyde, pyruvic acid, acetyl-CoA, and succinate—that may enter the Krebs cycle. AmnE also belongs to the YjgF/YER057c/UK114 family (also known as the Rid family), which is conserved in all domains of life and prefers structurally homotrimeric forms with diverse functional purposes. To study the mechanism of the modified meta-cleavage pathway in *Pseudomonas* sp. *AP–3*, we determined the first crystal structure of AmnE from *Pseudomonas* sp. *AP–3* at 1.75 Å. AmnE forms a unique homohexamer instead of a trimer which is normally adopted by the members of YjgF/YER057c/UK114 family. Based on the structure of the AmnE hexamer, we observed a hydrophobic base composed of six Lp3 loops (residues 122–131) in each of the AmnE protomers that have pivotal roles in the assembly of the hexamer. Eighteen hydrogen bonds formed by the residues Met^96^, Pro^126^, and Arg^56^, which surround the hydrophobic base, allowed the combination of the two trimers into a stable hexamer. The single mutant of AmnE R56A lost the ability to maintain the hexameric conformation, and revealed that the hydrogen bonds between residues Arg^56^ and Met^96^ have pivotal roles in the AmnE hexameric assembly.

## Introduction

**FIGURE 1:**
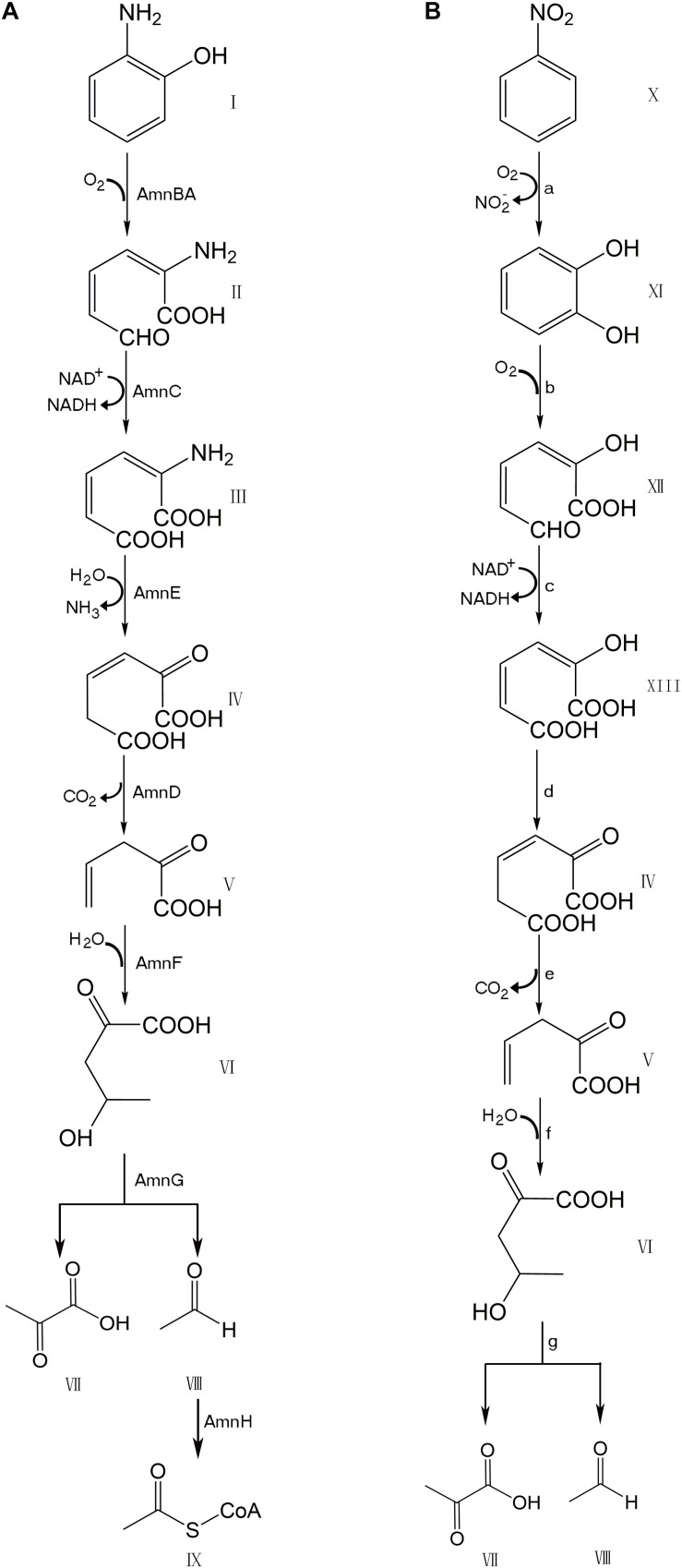
Comparison of modified meta-cleavage pathway in *Pseudomonas species AP-3* and meta-cleavage pathway in *Comamonas species JS765*. **(A)** Modified meta-cleavage pathway in *Pseudomonas species AP-3*. AmnBA: 2-aminophenol 1,6-dioxygenase. AmnC: 2-aminomuconic 6-semialdehyde dehydrogenase. AmnE: 2-aminomuconate deaminase. AmnD: 4-oxalocrotonate decarboxylase. AmnF: 2-oxopent-4-enoate hydratase. AmnG: 4-hydroxy-2-oxovalerate aldolase. AmnH: acetaldehyde dehydrogenase. **(B)** Meta-cleavage pathway in *Comamonas species JS765*. a: nitrobenzene dioxygenase. b: catechol 2,3-dioxygenase. c:2-hydroxymuconic semialdehyde dehydrogenase. d: 4-oxalocrotonate tautomerase. e:4-oxalocrotonate decarboxylase. f: 2-oxo-4-pentenoate hydratase. g:4-hydroxy-2-oxovalerate aldolase. I: 2-aminophenol. II: 2-aminomuconic 6-semialdehyde. III: 2-aminomuconic acid. IV: 2-oxo-3-hexene-1,6-dioate. V: 2-oxopent-4-enoic acid. VI: 4-hydroxy-2-oxovalerate. VII: Pyruvic acid. VIII: Acetaldehyde. IX: Acetyl-CoA. X: nitrobenzene. XI: catechol. XII: 2-hydroxymuconic semialdehyde. XIII: 2-hydroxymuconate. The figure was produced with ChemDraw Professional 15.1.

Many bacteria, including numerous species of *Pseudomonas*, possess genes for the degradation of aromatic compounds through the meta-cleavage pathway. *Pseudomonas species AP–3* (*Pseudomonas* sp. *AP–3*) was isolated by growth based on the use of 2*–*aminophenol as the sole carbon, nitrogen, and energy source (Takenaka et al., 1997). A modified meta-cleavage pathway for 2–aminophenol metabolism has been studied comprehensively. This pathway is similar to that known for the meta-cleavage of catechol, except that one of the hydroxyl groups on the metabolites is replaced by an amino group (Takenaka et al., 1998). The 2–aminophenol is degraded by *Pseudomonas* sp. *AP–3* via 2*-*aminomuconate*-*6-semialdehyde to 4-hydroxy-2-oxovalerate, which is further degraded to pyruvate and acetyl-CoA via the modified meta-cleavage pathway (Figure 1). A 13.9 kb region of the *AP–3* strain encompasses eight tight clusters of genes designed in the form of *amn[A-H].* Each gene in the modified meta-cleavage pathway is similar to the corresponding gene that operated in the meta-cleavage pathway, except for the *amnE* gene. The *amnE* gene product AmnE is a deaminase, and hydrolyzes 2-aminomuconate to 4-oxalocrotonate and releases ammonia in the modified meta-cleavage pathway (Takenaka et al., 2000). With the inclusion of the AmnE, the metabolic pathways of 2-aminophenol and its derivatives in *Pseudomonas* sp. *AP*–*3* consist of seven major enzymes translated from the *amn* gene cluster (*amnA–amnH*) into a single operon, including 2-aminophenol 1,6-dioxygenase (AmnBA), 2-aminomuconic 6-semialdehyde dehydrogenase (AmnC), 4-oxalocrotonate decarboxylase (AmnD), 2-aminomuconate deaminase (AmnE), 2-oxopent-4-enoate hydratase (AmnF), 4-hydroxy-2-oxovalerate aldolase (AmnG), and acetaldehyde dehydrogenase (AmnH). Zhongqi H. and Jim C. S. also revealed the mechanism of the deamination reaction in the modified meta-pathway and identified the 2-aminomuconate deaminase acted specifically on the unsaturated α-amino acid 2-aminomuconate in *P. pseudoalcaligenes JS45* (He and Spain, 1998). The deduced amino acid sequence of 2-aminomuconate deaminase in *Pseudomonas* sp. *AP–3* shows that AmnE belongs to a member of the YjgF/YER057c/UK114 protein family.

The YjgF/YER057c/UK114 protein family (also known as the Rid family) includes 200 prokaryotic and eukaryotic proteins, and consists of 130 amino acid residues (∼15 kDa) which are found in all three domains of life (Kim et al., 2001). Phylogenetic analysis divided this family into a widely distributed archetypal RidA subfamily, and into seven other subfamilies (Rid1–Rid7). The archetypal RidA subfamily is found in all domains, while the Rid1–7 subfamilies are present only in prokaryotes. These proteins have widespread roles in metabolic processes in nearly every organism, and some species encode multiple members (Niehaus et al., 2015). In bacteria and yeast, protein family members exhibit predefined roles in the biosynthesis of essential compounds. Conversely, the mammalian members involved in the regulation of cellular differentiation inhibit translations by degrading messenger ribonucleic acid (Schmiedeknecht et al., 1996). After decades of research, 34 structures, including AmnE in the RidA subfamily, have been identified and deposited in the protein data bank (PDB). Many other existing homologs have not been published yet. 31 structures in the RidA subfamily exhibit the preferential adoption of homotrimers and form a distinct inter-subunit cavity that may serve as a small molecular binding site, but with unknown functions. Based on the experimental evidence, the RidA subfamily members have been recently annotated as imine deaminase (Hodge-Hanson and Downs, 2017).

Although the 2-aminophenol metabolic pathway and the corresponding catabolic enzymes have been studied extensively, the knowledge of the structural basis of the catabolic enzymes is still lacking. In this study, we demonstrate that AmnE self-assembles in a unique homo-hexameric form that is different from a regular trimer model of the RidA subfamily. The homohexameric structure of AmnE has been determined at a high resolution (equal to 1.75 Å), and constitutes the first protein structure in the metabolic pathway of 2-aminophenol. We analyzed the structure of the AmnE hexamer and explained how the six identical protomers assembled into a stable homohexameric conformation. Meanwhile, we identified that the residues Ala^49^–Pro^59^ on the Lp2 loop region (residues 38–60) of AmnE have pivotal roles in the assembly of the hexameric structure. The single mutant of AmnE R56A lost the ability to maintain the hexameric conformation, and thus revealed that the hydrogen bonds between residues Arg^56^ and Met^96^ have pivotal roles in the AmnE hexameric assembly.

## Materials and Methods

### Generation of Wild Type and Correlated Mutant Plasmid Constructs

The gene encoding deaminase AmnE from *Pseudomonas* sp. *AP–3* was synthesized by Sangon Biotech Co., Ltd., China, and was cloned into a pET–28a (GE Healthcare, United States) using a fast cloning method (Li et al., 2011). The single mutants AmnE R56A and AmnE P126A, and the truncated mutant AmnEΔ49–59 were generated by designing primers that encoded the mutated amino acids. Mutagenetic constructs were confirmed by commercial deoxyribonucleic acid (DNA) sequencing (Sangon Biotech, China). The primers used in this study are listed in Table 1.

**TABLE 1:** Primers used in this study.

**Gene**	**Primer**	**Sequence (5′–3′)**
AmnE	HF	CTGGTGCCGCGCGGCAGCATGGTTAGCAAA GCTGATAA
AmnE	HR	GATGATGATTCCTCCTCCTTAGAGCGGCT TGTATGCCA
AmnEΔ49–59	F	AATACGTTTGTGGGCAATATTGAGC
AmnEΔ49–59	R	GCCCACAAACGTATTATCGGGCCTG
AmnE R56A	F	CCGGATGATACCGGTGCACCCAGGCCA
AmnE R56A	R	CGACCGGTATCATCCGGTTCAGCGCCC
AmnE P126A	F	GCCGTCCATCAACTGGCGCACCCTCAAT
AmnE P126A	R	CGCCAGTTGATGGACGGCGACCGTGGTA

### Protein Expression and Purification

Wild plasmids of the type AmnE and correlated mutants were transformed into the BL21 (DE3) pLysS strain (TransGen Biotech, China) and grown in Luria-Bertani (LB) medium containing kanamycin (50 μg/ml) and chloromycetin (34 μg/ml). Growing cells were induced at an optical density (OD_600_) value in the range of 0.6–0.8 by the addition of 0.5 mM isopropyl-β-D-1-thiogalactopyranoside (IPTG) for 16 h at 16°C. Cells harvested by centrifugation for 10 min at 4,000 revolutions per minute (rpm) were suspended in lysis buffer (20 mM Tris, 200 mM NaCl, and 10 mM imidazole, pH 8.0) and lysed using a high-pressure homogenizer (JNBIO, China) at 4°C. The cell lysate was centrifuged for 30 min at 15,000 rpm at 4°C (Thermo Sorvall LYNX 6000, United States), and the supernatant was loaded onto a Ni–NTA affinity chromatography column (GE Healthcare, United States) which was equilibrated with a lysis buffer. The unbound proteins were washed away with wash buffer (20 mM Tris, 200 mM NaCl, 50 mM imidazole, pH 8.0). The tightly bound proteins, which mainly comprised AmnE, were eluted with elution buffer (20 mM Tris, 200 mM NaCl, and 250 mM imidazole pH 8.0). The N-terminal 6 × His tag was cleaved with Thrombin (Sigma, United States) in the dialysis buffer (20 mM Tris, 20 mM NaCl, and 5% (v/v) glycerol, pH 8.0) at 6°C overnight. The AmnE protein was further purified by anion-exchange chromatography on a Resource Q column (GE Healthcare, United States). The protein was then purified by size-exclusion chromatography using a Superdex75 10/300 GL column (GE Healthcare, United States). The single mutants AmnE R56A and AmnE P126A, and the truncated mutant AmnEΔ49–59 were pre-equilibrated with buffer (50 mM HEPES, 150 mM NaCl, 1 mM DTT, 1 mM EDTA, and 5% (v/v) glycerol, pH 8.0). All proteins were concentrated by ultrafiltration using a Millipore centrifugal ultrafiltration device (Amicon Ultra, 3 kDa cutoff, Germany) and analyzed using sodium dodecyl sulfate polyacrylamide electrophoresis (SDS–PAGE) (Supplementary Figures S1–S4). For crystallization purposes, the protein AmnE was concentrated to 10 mg/ml.

### Crystallization, X-Ray Data Collection, and Structure Determination

A Gryphon robot (Art Robbins Instruments, United States) was used for the initial screening of 96 well plates at 20°C with the sitting-drop vapor diffusion method. Drops were prepared by mixing 0.5 μl of purified protein and 0.5 μl of reservoir solution. Commercial crystallization kits (Crystal Screen, Crystal Screen 2, Index Screen, Salt screen, and PEG Screen) were bought from Hampton Research (United States). Protein crystals were obtained only at 0.2 M MgCl_2_•6 H_2_O, 0.1 M Tris–HCl pH 8.5, 30% (w/v) PEG 4000 (Supplementary Figure S5A). Optimization was carried out in 24-well plates by using hanging-drop method. Drops were mixed with 1 μl of protein solution at 10 mg/ml and 1 μl of reservoir solution. The high-quality crystals were obtained in 0.1 M CAPSO [3-(Cyclohexylamino)-2-hydroxy-1-propanesulfonic acid] pH 9.6, 0.2 M MgCl_2_•6 H_2_O, and 30% (w/v) PEG 4000 (Supplementary Figure S5B). The crystals were flash-cooled in liquid nitrogen and the diffraction data were collected in the Shanghai Synchrotron Radiation Facility (SSRF) Beamline BL19U1 (Shanghai, China). The data were processed with HKL2000 software (Otwinowski and Minor, 1997). The best crystal diffracted X-rays and formed a pattern with a high-resolution equal to 1.75 Å. The space group of AmnE is C121, with cell dimensions set at *a* = 170.871 Å, *b* = 54.781 Å, *c* = 134.594 Å (Supplementary Figure S5C), while the AmnE structure was solved by molecular replacement method with the Phaser (Lebedev et al., 2008) in the CCP4 suite (Winn et al., 2011), using the initial model of *Pyrococcus horikoshii*-PH0854 (PDB: 2DYY). The refined model was further built manually according to the electron density map with COOT (Emsley and Cowtan, 2004), and the qualities of the refined models were assessed with PHENIX (Adams et al., 2010). The final structure was derived to a R_*work*_ factor of 15.7% (R_*free*_ factor of 19.5%). All figures were generated by using PyMOL^1^. Data collection, statistics, and structure-refinement statistical outcomes are listed in Table 2.

**TABLE 2:** Data-collection and relevant statistics (values in parentheses correspond to the shell with the highest resolution).

	**AmnE**
Data collection	
Wavelength (Å)	0.9789
Beamline	BL19U1
Detector	CCD Pilatus CBF
Space group	C121
Unit-cell parameters (Å,°)	a = 170.871, b = 54.781, c = 134.594; α = 90.00, β = 101.89, γ = 90.00.
Resolution (Å)	50.00–1.76 (1.76–1.81)
R_*merge*_(%)^†^	90.1 (53.5)
Average *I/*σ*(I)*	28.25 (1.94)
No. of observed reflections	234852 (19456)
No. of unique reflections	120742 (9970)
Completeness (%)	99.3 (99.0)
Redundancy	3.4 (3.3)
Matthews coefficient (Å^3^Da^–1^)	2.32
Solvent content (%)	47
Molecules per asymmetric unit	9
Refinement	
Resolution (Å)	49.511–1.754
R_*work*_/R_*free*_	0.1570/0.1949
Ramachandran favored (%)	96.56
Ramachandran allowed (%)	3.44
Ramachandran outliers (%)	0.00
No. of atoms	
Protein	9628
Ligand	2
Water	1042
Wilson B value	22.69
Root-mean-square deviations	
Bond lengths (Å)	0.007
Bond angles (°)	0.980

*^†^R_merge_ = ∑_hkl_ ∑_i_|I_i_(hkl)−⟨I(hkl)⟩|/∑_hkl_ ∑_i_I_i_(hkl), where I_i_(hkl) is an individual intensity measurement and ⟨I(hkl)⟩ is the average intensity for all reflections.*

### Dynamic Light Scattering

Measurements were obtained using a Zetasizer μV (Malvern Panalytical, Worcestershire, United Kingdom) with AmnE at a concentration of 10 mg/ml in a buffer solution [50 mM HEPES, 150 mM NaCl, 1 mM DTT, 1 mM EDTA, and 5% (v/v) glycerol, pH 8.0] at 4°C, 16°C, 20°C, and 25°C, separately. The results were analyzed using software provided by the manufacturer. Experimental errors were estimated based on the estimated standard deviations.

### Analytic Size-Exclusion Chromatography

Analytic size-exclusion chromatography (SEC) was adopted to investigate the oligomeric state of AmnE and associated mutants using a Superdex 200 10/300 GL column (GE Healthcare, United States). The column was pre-equilibrated with an analysis buffer [50 mM HEPES, 150 mM NaCl, 1 mM DTT, 1 mM EDTA, and 5% (v/v) glycerol, pH 8.0]. Furthermore, all the samples, at a concentration of 1 mg/ml, were analyzed at a flow rate of 0.3 ml/min at 16°C. The molecular mass was calculated using the following equation,

$$V\,e=-b^{\prime }\,\text{lg}\,M\,r+c^{\prime }$$

*Ve*: the volume at which the intermediate molecules elute; *Mr*: molecular mass.

### Chemical Cross-Linking Assay

AmnE wild type and mutant proteins were purified with the use of a Superdex 75 10/300 GL column (GE Healthcare, United States). Glutaraldehyde (25%) (Sigma, United States) was diluted at various concentrations (0.1, 0.2, 0.3, 0.4, 0.5, 0.6, 0.7, 0.8, 0.9, and 1%) in distilled water. Proteins in buffer [50 mM HEPES, 150 mM NaCl, 1 mM DTT, 1 mM EDTA, and 5% (v/v) glycerol, pH 8.0] were incubated with glutaraldehyde at 25°C for 30 min. The reaction was then quenched with the addition of 10 μl solution of 1 M Tris–HCl (pH 8.0), and 5 × SDS–PAGE loading buffer. All samples were analyzed on SDS–PAGE gels.

### CD Spectroscopy

Before CD measurements, all proteins were purified using a Superdex 75 10/300 GL column (GE Healthcare, United States) in buffer [50 mM HEPES, 150 mM NaCl, 1 mM DTT, 1 mM EDTA, 5% (v/v) glycerol, pH 8.0]. The CD spectra were recorded on a Jasco J–715 Spectropolarimeter (JASCO, MD, United States) with the use of three scans on average within the 190–260 nm wavelength range from solutions in 10 mm path length quartz cuvettes at 25°C. Raw ellipticity data θ_*obs*_ (in 10^4^ millidegrees) were converted to mean residue ellipticity (θ) (in 10^4^ millidegrees) using the formula,

$$\left(\theta \right)=\frac{\left(\theta \,o\,b\,s\times M\,R\,W\times 100\right)}{\left(c\times l\right)}$$

where θ is the ellipticity in 10^4^ millidegrees, *l* is the path length of the cuvette in cm, *MRW* is the mean residue weight, and *c* is the concentration in mg/ml. Data were analyzed with Microsoft Office Excel and Origin 8.

## Results

### Protomeric Structure of AmnE

**FIGURE 2:**
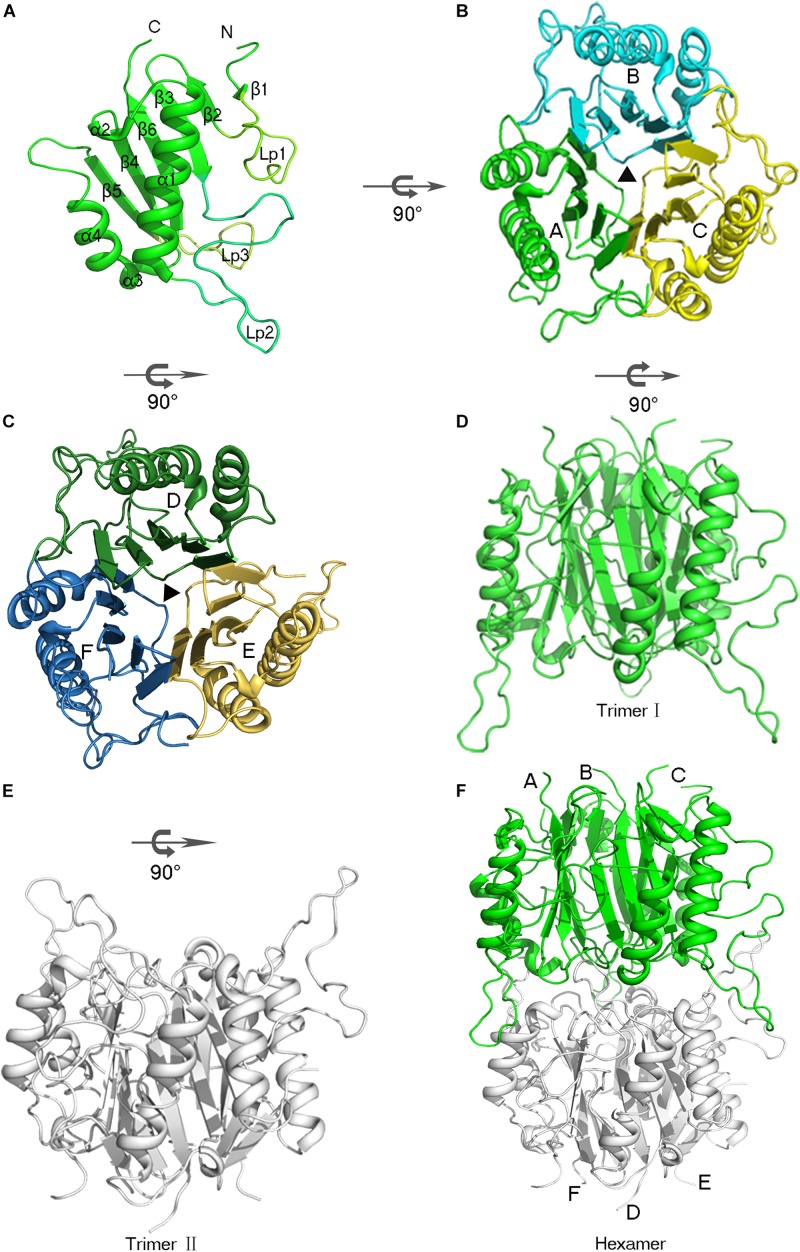
Overview of the hexameric assembly of AmnE. **(A)** Three-dimensional view of the AmnE protomer. The ribbon diagram is colored in green. **(B)** AmnE trimer I (A/B/C). Subunits are drawn in different colors in this ribbon diagram viewed along the threefold axis. Chains A–C are respectively, colored in green, cyan, and yellow. **(C)** AmnE timer II (D/E/F). Subunits are drawn in different colors in this ribbon diagram viewed along the threefold axis. Chains D–F are respectively, colored in forest green, yellow orange, and sky-blue. **(D–F)** The AmnE hexamer is composed of two identical trimers (I and II). Trimer I is colored in green and trimer II is colored in gray. The figure was produced with PyMOL (http://www.pymol.org).

The AmnE protomer is a 142 residues polypeptide folded into a single domain. The overall structure of the AmnE protomer shows an α + β fold with a β-sheet (β_1_ - β_6_), two α-helices (α_1_, α_4_), and two 3_10_-helices (α_2_, α_3_), in an arrangement of β_1_ (residues 9 –10), β_2_ (residues 25–28), β_3_ (residues 31–37), α_1_ (residues 61 –78), α_2_ (residues 83–85), β_4_ (residues 86–93), β_3_ (residues 96–98), α_4_ (residues 99–106), β_5_ (residues 116–121), and β_6_ (residues 132–139) (Figure 2A). Six β-strands are aligned in a single sheet, and two long α-helices (α_1_, α_4_) are arranged in an almost parallel orientation on one side of the β-sheet. Additionally, two 3_10_ helices are observed in the structure of which α_2_ is positioned at the end of α_1__,_ followed by the β-strand β_4_. Accordingly, α_3_ is located at the N-terminus of the α-helix (α_4_). In the β-sheet, β_4_ and β_5_ are parallel to each other, and all other strands are antiparallel. Three large loops (Lp1–3) extend from one side of the β-sheet consisting of an unfolded region. The Lp1 (residues 11–24) and Lp3 (residues 122–131) are located separately between the two β-strands, (β_1_, β_2_) and (β_5_, β_6_). Lp2 (residues 38–60) is the longest loop and connects β_3_ and α_1_ (Figure 2A).

The structure of the AmnE protomer is highly conserved in the YjgF/YER057c/UK114 family. The DALI search from the PDB database retrieved 50 homologs with similar structural forms in the case of the AmnE protomer. Three protein structures are chosen to represent all prokaryotic and eukaryotic proteins in this protein family. They include the human trichloroacetic acid-soluble protein p14.5 (hp14.5, PDB code: 1ONI), the *E. coli* TdcF protein (PDB code: 2UYN), and YEO7 in *Saccharomyces cerevisiae* (PDB code: 1JD1). The protomers of the three proteins are highly conserved structurally (Supplementary Figures S6A–C). Pairwise superposition of the three protomers yield root-mean-square deviation (RMSD) values equal to 0.682 Å (for 84 Cα atoms, AmnE/TdcF), 0.702 Å (for 85 Cα atoms, AmnE/YE07), and 0.850 Å (for 79 Cα atoms, AmnE/hp14.5). The region that exhibits considerable structural protein diversity is located in the unfolding region. The Lp2 of AmnE is along the disorder loop, and extends from the main body compared to the other three structures. The sequence alignment data revealed that residues Ala^49^–Pro^59^ are in a unique region in AmnE, which is deficient in Tdcf, YE07, and p14.5 (Supplementary Figure S6G).

### Structural Basis of AmnE Trimer

**FIGURE 3:**
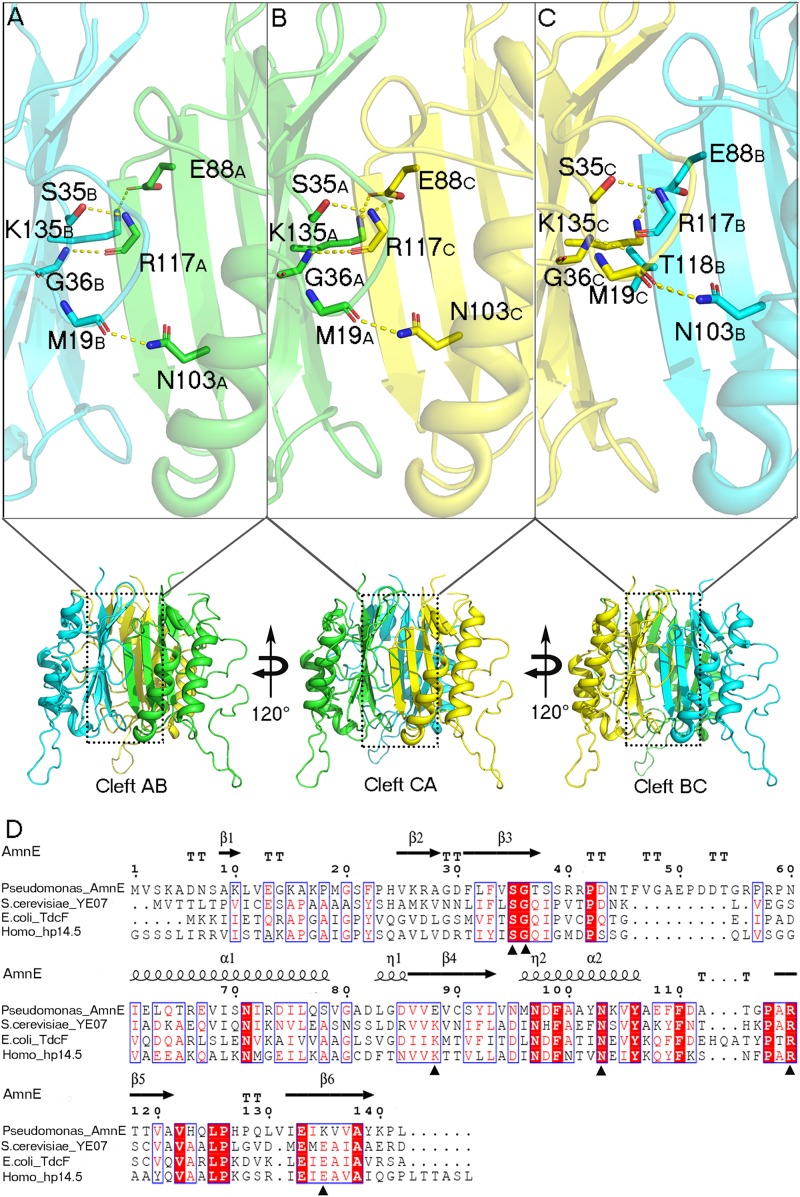
Analyses of AmnE trimeric clefts. Conserved residues Ser^35^, Gly^36^, Glu^88^, Gln^103^, Arg^117^, and Lys^135^, are highlighted in the three clefts with sticks. **(A)** Interface of cleft AB. **(B)** Interface of cleft CA. **(C)** Interface of cleft BC. **(D)** Comparison of AmnE with the other three members of the YjgF/YER057c/UK114 family. Sequence alignment of AmnE from *Pseudomonas* sp. *AP–3* with YE07 from *Saccharomyces cerevisiae* (34.00% sequence identity), TdcF from *Escherichia coli* (29.00% sequence identity), and hp14.5 from *homo sapiens* (28.72% sequence identity). Residues labeled with triangles are conserved residues involved in the ligand binding pocket. Sequences were aligned using the tool obtained from the web site (https://www.ebi.ac.uk/Tools/msa/clustalo/) and the alignment was presented using the online ESPript 3.0 server (http://espript.ibcp.fr/ESPript/ESPript/).

AmnE adopts the classic homotrimer formed by three protomers like the other members of the YjgF/YER057c/UK114 family (Figure 2B). The core of the homotrimer is composed of three β-sheets. Each one of these is closed into an empty triangular barrel-type structure and is surrounded by six α-helices on its outer part. The inner surface of the barrel forms a highly hydrophobic environment composed of β3, β4, and β6 strands. Viewed from the top to the bottom of the trimer, one end of the barrel is sealed off by three phenylalanines (Phe^31^), while the other is sealed by a hydrophobic loop Lp3 from each of the three protomers (Supplementary Figure S7). Residues Leu^32^, Val^86^, Val^87^, Leu^93^, Ile^132^, and Ile^134^ on the inner surface of the barrel are conserved in all of the members of the RidA subfamily (Supplementary Figure S8A). The hydrophobic residues are distributed on the inner surface of the barrel to maintain the stability of the homotrimer of AmnE. The single protomer-accessible surface buried in the trimer is 1994.1 Å^2^, and the proportion of the buried area to a protomer’ total surface area is 25.89%. On the outer parts of the trimer, the interfaces of the adjacent subunits form three equivalent clefts, i.e., AB, BC, and CA (Figures 3A–C). These clefts are formed by α_4_, β_4_, and β_5_ in one subunit, and β_2_, β_3_, and β_6_, loop Lp1, and α_4_ in the adjacent subunit. Lp1 covers the cleft and is associated with elevated temperature factors and increased RMSD values among the three protomers. Comparison of the protein–ligand complex structures of hp14.5 (PDB code: 1ONI) and TdcF (PDB code: 2UYN), shows that the Lp1 of AmnE is potentially involved in unknown ligand binding (Supplementary Figure S9). The residues Ser^35^, Gly^36^, Glu^88^, Gln^103^, Arg^117^, and Lys^135^ in these three clefts are highly conserved in the YjgF/YER057c/UK114 family (Figure 3D). The areas of the three clefts (AB, BC, and CA) in AmnE were respectively, calculated and were equal to 760.43, 760.47, and 741.33 Å^2^, respectively. These areas could provide ideal contact surfaces for small molecules with unknown functions. The DALI search from the PDB library lists 26 homologies of the AmnE trimer in YjgF/YER057c/UK114 family (Table 3). The structural superimposition suggests that the trimer is conserved in YjgF/YER057c/UK114 family proteins. The RMSD value estimated based on 192–290 Cα atoms in these structures varied in the range of 0.67–1.89 Å, with a sequence identity in the range of 23.00–40.51%. The superposing structures revealed that three Lp2 loops from each AmnE protomer extended out far from the main body of the trimer, and formed a unique conformation compared to other homologs in the YjgF/YER057c/UK114 family (Supplementary Figures S6D–F). Accordingly, the shape of the AmnE trimer resembled a three-legged structure.

**TABLE 3:** Homologous proteins of AmnE trimer.

**PDB code**	**Species**	**Cα atoms**	**RMSD**	**Sequence identities**
6IZH	*Pseudomonas* sp. *AP*–*3*	423	0.000	100%
5YU2	*Staphylococcus aureus*	271	0.672	28.33%
1JD1	*Saccharomyces cerevisiae*	279	0.691	34.00%
3I7T	*Mycobacterium tuberculosis*	260	0.721	32.00%
2B33	*Thermotoga maritima*	263	0.735	33.06%
2UYP	*Escherichia coli*	252	0.760	29.00%
2UYK	*Escherichia coli*	251	0.767	29.00%
3L7Q	*streptococcus mutans*	274	0.800	34.71%
1NQ3	*Capra hircus*	257	0.800	35.06%
1QAH	*Rattus norvegicus*	270	0.807	28.00%
1QD9	*Bacillus subtilis (strain 168)*	252	0.830	40.51%
1XRG	*Clostridium thermocellum*	262	0.842	31.97%
5Y6U	*Bacillus subtilis (natto)*	254	0.849	40.51%
1QU9	*Escherichia coli*	238	0.855	29.93%
3MQW	*Entamoeba histolytica*	252	0.893	34.18%
1ONI	*Homo sapiens*	247	0.900	28.72%
3V4D	*Escherichia coli*	290	0.970	28.00%
2DYY	*Pyrococcus horikoshii*	267	0.970	39.23%
3K0T	*Pseudomonas syringae*	260	0.991	25.00%
1PF5	*Escherichia coli (strain K12)*	276	1.066	32.00%
5V4F	*Yersinia pestis*	262	1.163	28.00%
2CWJ	*Aeropyrum pernix*	271	1.173	30.00%
3LME	*Rhodopseudomonas palustris*	245	1.290	23.00%
1J7H	*Haemophilus influenzae*	245	1.332	29.00%
3K12	*Pseudomonas aeruginosa*	224	1.337	32.32%
3GTZ	*Salmonella typhimurium*	243	1.397	32.00%
3I3F	*Giardia lamblia*	192	1.889	32.00%

### The Structure of AmnE Hexamer

AmnE exists as a hexamer in solution and not in a trimeric state like the other members of the YjgF/YER057c/UK114 family. We identified the hexameric form of AmnE with the use of size-exclusion chromatography and cross-linking methods. AmnE was eluted at 13.36 ml on a Superdex 200 size-exclusion column, and its calculated molecular mass was 103.5 kDa, which was close to that of the hexamer. This finding was also supported by crosslinking data. The crystal structure of AmnE was solved in the space group C121 with nine protomers (A, B, C, D, E, F, G, H, I) composed of three identical homotrimers (trimers I, II, and III) in one asymmetric unit. One of the AmnE hexamers is organized based on the use of two identical trimers, trimer I (A/B/C) and trimer II (D/E/F) (Figures 2B–F). Trimer III forms a hexamer with the use of trimer III in the neighboring asymmetric unit in the crystal lattice. The quaternary structure of AmnE was analyzed by PDBePISA. The total accessible surface of the two trimers buried in the hexamer is very large and equal to 3954.1 Å^2^. Correspondingly, the proportion of the buried area to the total area of the two trimers is 11.59%.

**FIGURE 4:**
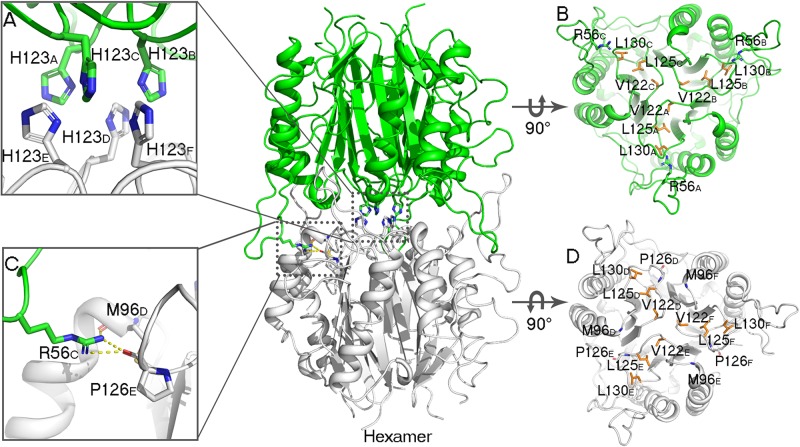
Analyses of AmnE hexameric interface. **(A)** Details of six His^123^ located on the Lp3 are shown using sticks. **(B)** Hydrophobic loop Lp3 in trimeric structures of AmnE, the hydrophobic residues (Val^122^, Leu^125^, and Leu^130^) are shown in orange sticks. The three residues of Arg^56^ that participate in hydrogen-bond formations in the hexameric structure are shown in green sticks. **(C)** Details of hydrogen bonds connecting Arg^56^ with Met^96^ and Pro^126^ are shown using sticks. **(D)** Hydrophobic loop Lp3 in the trimeric structures of AmnE and the hydrophobic residues (Val^122^, Leu^125^, and Leu^130^) are shown in orange sticks. The residues of Met^96^ and Pro^126^ that participate in hydrogen bond formations in the hexameric structure are shown in white sticks. The figure was produced with PyMOL (http://www.pymol.org).

In the AmnE hexameric structure, two trimers (trimer I, timer II) interact in a back-to-back manner through the hydrophobic region composed of six Lp3 loops (Lp3_*A*_, Lp3_*B*_, and Lp3_*C*_, from trimer I, and Lp3_*D*_, Lp3_*E*_, Lp3_*F*_, from trimer II). These loops are used to seal off the bottom of the hydrophobic channel in all of the trimers. Six residues His^123^ (His^123^_*A*__–__*F*_), located on the loops Lp3, form a hydrophobic base with residues Val^122^_*A*__–__*F*_, Leu^125^_*A*__–__*F*_, and Leu^130^_*A*__–__*F*_ present in the AmnE hexamer (Figures 4A,B,D). Outside of the hexamer, six loops (Lp2_*A*_, Lp2_*B*_, and Lp2_*C*_, from trimer I, and Lp2_*D*_, Lp2_*E*_, and Lp2_*F*_, from trimer II) surround the hexamer and extend into the cleft area of the opposite trimer to form a solid football-like shape. Based on the structural analysis, the Lp2 (residues 49–59) was identified to play a pivotal role in the maintenance of the stability of the AmnE hexamer. There are six pivotal residues Arg^56^ (Arg^56^_*A*__–__*F*_) located on loops Lp2 (Lp2_*A*_–_*F*_) that formed eighteen hydrogen bonds in the hexamer with residues Pro^126^ (Pro^126^_*A*__–__*F*_) and Met^96^ (Met^96^_*A*__–__*F*_) (Figure 4C).

**FIGURE 5:**
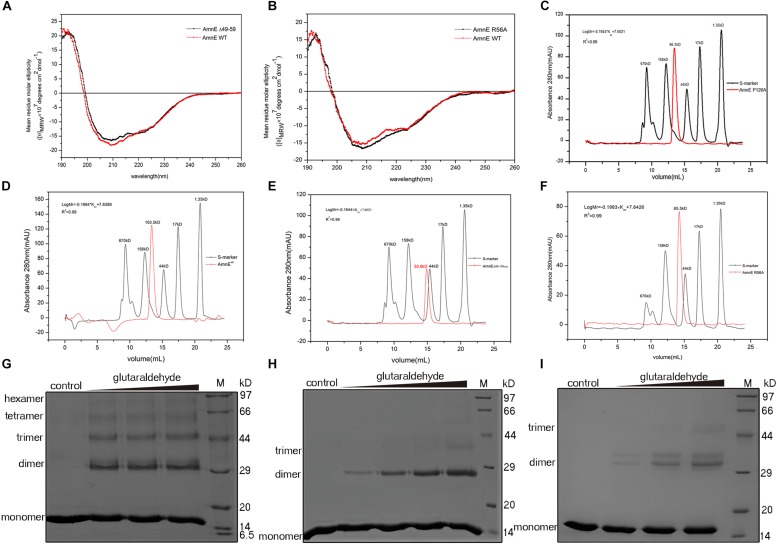
Biochemical characterization of AmnE hexamerization. **(A)** Measurement of AmnEΔ49–59 (black line) and wild-type AmnE (red line) based on CD spectroscopy. **(B)** Measurement of AmnE R56A (black line) and wild-type AmnE (red line) based on CD spectroscopy. **(C)** Analytic size-exclusion chromatography of mutant AmnE P126A. The red solid curve refers to the AmnE P126A and the black solid curve refers to the molecular weight standards. **(D)** Analytic size-exclusion chromatography of wild-type AmnE. The red solid curve refers to the AmnE and the black solid curve refers to the molecular weight standards. **(E)** Analytic size-exclusion chromatography of mutant AmnEΔ49–59. The red solid curve refers to AmnEΔ49–59 and the black solid curve refers to the molecular weight standards. **(F)** Analytic size-exclusion chromatography of mutant AmnE R56A. The red solid curve refers to AmnE R56A and the black solid curve refers to the molecular weight standards. **(G)** Crosslinking of wild-type AmnE with increasing concentrations of glutaraldehyde are shown on SDS–PAGE gel, untreated wild-type AmnE loaded as control. In this and subsequent subfigures, M denotes a protein marker. **(H)** Cross-linking assay used to detect the polymerization forms of mutant AmnEΔ49 - 59. Crosslinking of AmnEΔ49 - 59 with increasing concentration of glutaraldehyde are shown on SDS–PAGE gel, untreated AmnEΔ49 - 59 loaded as control. **(I)** Cross-linking assay used to detect the polymerization forms of mutant AmnE R56A. Crosslinking of AmnE R56A with increasing concentration of glutaraldehyde are shown on SDS–PAGE gel, untreated AmnE R56A loaded as control.

To further confirm that the functions of Lp2 and Arg^56^ contribute to the maintenance of the conformation of the AmnE hexamer *in vitro*, the mutant proteins AmnEΔLp2 (residues Ala^49^–Pro^59^) and AmnE R56A were prepared and measured with CD spectroscopy before subsequent experiments (Figures 5A,B). The oligomerization states of the wild type and mutants of AmnE were evaluated by SEC. The calculated molecular mass of the wild-type AmnE and mutant AmnE P126A were respectively equal to 103 and 96.3 kDa, and were close to the theoretical molecular mass of the hexamer (Figures 5C,D). However, AmnEΔLp2 (residues Ala^49^–Pro^59^) and R56A exhibited larger retention volumes, which corresponded to the molecular weight of the AmnE trimer (Figures 5E,F). Meanwhile, a glutaraldehyde crosslinking experiment was conducted to verify the multimeric state of each protein (Figures 5G–I). Primarily and foremost, we confirmed that the AmnE hexamer was in a stable state in solution, and that the residues Arg^56^ and Met^96^ located on the hexameric interface were two key residues required to maintain the hexameric conformation of AmnE.

## Discussion

The 2-aminophenol compounds are intermediates in the biodegradation of nitrobenzene. The benzene ring cleavage and deamination steps are essential in the mineralization of 2-aminophenol for the growth of the assimilating bacteria. The metabolic pathway for 2-aminophenol in *Pseudomonas* sp. *AP–3* is referred to as the modified meta-cleavage pathway, in which 2-aminomuconate is transformed into 4-oxalocrotonate by the deaminase AmnE (Takenaka et al., 2000). In this study, we elucidated the first structure of 2-aminomuconate deaminase involved in the 2-aminophenol pathway in *Pseudomonas* sp. *AP–3*. AmnE also belongs to a member of the RidA subfamily in the YjgF/YER057c/UK114 family. In this family, proteins are distributed in all domains of life, apparently in the archetypal RidA subfamily and in seven other subfamilies. Members of the RidA subfamily prefer to form the trimeric, barrel-like quaternary structures, and inter-subunit cavities with diverse biological roles. Many of these are poorly understood despite their widespread existence (Sinha et al., 1999). In the PDB library, there are 33 structures of RidA subfamily in total, including one nuclear magnetic resonance (NMR) structure and 32 crystal structures. Most of these structures have not been published or characterized yet by the authors. Therefore, we expect to analyze all of the structures in the RidA subfamily and reveal that there are two primary types of homomultimeric protein complexes, i.e., trimeric and hexameric forms.

**FIGURE 6:**
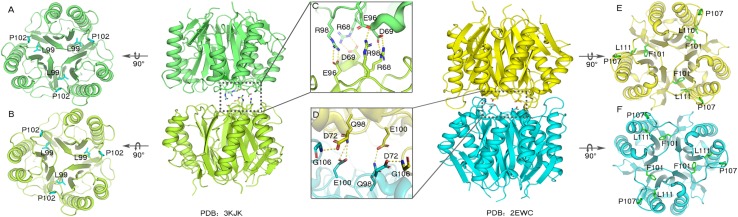
Analyses of Nmb1025 (PDB: 3KJK) and hypothetical protein SPy_2060 (PDB: 2EWC) hexameric structure interfaces. **(A,B)** Hydrophobic loop Lp3 in the trimeric structures of Nmb1025 in which the hydrophobic residues (Leu^99^ and Pro^102^) are shown using blue sticks. **(C)** Details of hydrogen bond formation of hexameric Nmb1025. The participating residues (Arg^68^, Asp^69^, Glu^96^, and Arg^98^) are highlighted with sticks. **(D)** Details of hydrogen bond formation of hexameric protein SPy_2060. Participating residues (Asp^72^, Gln^98^, Glu^100^, and Gly^106^) are highlighted as sticks. **(E,F)** Hydrophobic loop Lp3 in trimeric structures of protein SPy_2060, whereby the hydrophobic residues (Phe^101^, Pro^107^, and Leu^111^) are shown in green sticks. The figure was produced with PyMOL (http://www.pymol.org).

The trimeric conformation adopted by the RidA subfamily proteins is structurally conserved, and even the sequence similarity is comparatively low. These proteins adopt homotrimeric forms, and the clefts between the protomeric subunits are suggested to have functional relevance (Volz, 1999; Deaconescu et al., 2002; Deriu et al., 2003). The alignment of the RidA subfamily proteins has identified several residues in AmnE, including Ser^35^, Gly^36^, Glu^88^, Gln^103^, Arg^117^, and Lys^135^, which are highly conserved in clefts located at the subunit interfaces of the trimer (Supplementary Figure S8A). This indicates that these proteins play a similar role in cells. Lambrecht et al. revealed that RidA subfamily proteins exhibit enamine/imine deaminase activity and accelerate the release of ammonia from reactive enamine/imine intermediates (Lambrecht et al., 2010). Parsons et al. identified several compounds that interacted separately with the RidA subfamily protein HI0719 and protein hp14.5 in a pocket located in the clefts of the trimer separately (Parsons et al., 2003; Manjasetty et al., 2004). Therefore, the trimerization of the RidA subfamily proteins is necessary for achieving proper functionality *in vivo*. However, AmnE adopts homohexameric forms both in crystal structures and in solution states. A DALI search in the PDB library retrieved two similar hexameric structures (PDB code: 3KJK, 2EWC) based on the structure of the AmnE hexamer. These two proteins were identified as Nmb1025 in *Neisseria Meningitidis* and SPy_2060 in *Streptococcus Pyogenes M1 Gas*. All three proteins adopted hexameric assembly, which comprised two identical trimers in a back-to-back configuration forming the hydrophobic base in the middle, and six hydrophobic loops in the center of the hexamer (Figures 6A,B,E,F). Meanwhile, 15–21 hydrogen bonds around the hydrophobic base connected the two trimers together and maintained a stable hexameric structure. The hexameric structures of the proteins Nmb1025, SPy_2060, and AmnE, were analyzed by PDBePISA (Supplementary Table S1). All three proteins formed a hydrophobic base in loop Lp3 that was composed of the highly conserved residues Val^122^, Leu^125^, Pro^126^, Pro^128^, and Leu^130^ on the AmnE loop Lp3, as verified by sequence alignment in RidA subfamily (Supplementary Figure S8A). In addition to the hydrophobic base in the center of the hexamer, the Nmb1025 hexamer recruited residues Arg^68^, Asp^69^, Glu^96^, and Arg^98^ from each of the protomers, form hydrogen bonds (Figure 6C). The structures of the hypothetical protein Spy_2060 formed five hydrogen bonds based on the Asp^72^, Gln^98^, Glu^100^, and Gly^106^ from each of the protomers (Figure 6D). Therefore, the stable hexamer requires the hydrophobic base with 15–21 hydrogen bonds around it to connect the two trimers together. However, these residues forming the hydrogen bonds in three hexameric structures are not highly conserved in RidA subfamily. This is probably the reason why most of members in RidA subfamily adopt a trimeric form instead of hexameric conformation. We constructed a multigene expression system including *amnA, amnB*, and *amnC* genes from *Pseudomonas* sp. *AP–3* and expressed in *E. coli* to produce 2-aminomuconate in order to test the enzyme activity of hexameric AmnE and trimeric variants due to lack of stable substrate and cells of *Pseudomonas* sp. *AP–3*. However, we unable to get the 2-aminomuconate due to its high instability. Therefore, the hexameric structure of AmnE presented is the first step to understanding the molecular mechanism of the different oligomerization types and their relation to their enzyme activity or substrate specificity in the modified meta-cleavage pathway.

## Data Availability

Protein Data Bank Accession Codes: the atomic coordinates and structural factors of AmnE were submitted to the RCSB Protein Data Bank. The accession code is 6IZH.

## Author Contributions

DS and YA-C conceived the experimental study, designed the experiments, analyzed the data, and wrote the manuscript. HJ and DL designed the fast-cloning protocol and assisted YA-C in the preparation of the expression vectors. YA-C and TH expressed and purified the wild type and mutants of AmnE. YA-C crystallized AmnE. YA-C and YI-C collected the X-ray diffraction data. DS and YI-C determined and analyzed the crystal structures. YA-C measured the multimerization of the AmnE and mutants by cross-linking and gel-filtration experiments. YR, BY, GB, and HD provided the material, helped to design the experiments, and analyzed data. DS reviewed and provided the approval for the submission/publication of the manuscript.

## Conflict of Interest Statement

The authors declare that the research was conducted in the absence of any commercial or financial relationships that could be construed as a potential conflict of interest.

## Footnotes

1 http://www.pymol.org

## Abbreviations

AmnE: 2-aminomuconate deaminase
SEC: size-exclusion chromatography.
