# Supplementary material for: A Unique Homo-Hexameric Structure of 2-Aminomuconate Deaminase in the Bacterium Pseudomonas species AP–3
Source: Front Microbiol. 2019 Sep 6;10:2079. doi: 10.3389/fmicb.2019.02079 (PMC6742747; doi:10.3389/fmicb.2019.02079)
Supplement: Supplementary file 1 [file Data_Sheet_1.pdf]

Supplementary Material

Supplementary table 1.Hydrogen bonds on the interface of the three hexameric structures

PDB code	Structure 1	Dist. [Å]	Structure 2
6IZH	C: Arg 56[NH2]	3.57	D: Met 96[O]
	C: Arg 56[NH1]	2.98	E: Pro 126[O]
	C: Arg 56[NH2]	2.80	E: Pro 126[O]
3KJK	B: Asp69[OD1]	2.78	E: Arg 68[NE]
	B: Asp 69[OD2]	2.70	E: Arg 68[NH2]
	B: Glu 96[OE2]	2.55	E: Arg 98[NH2]
	B: Arg 68[NH2]	2.63	E: Asp 69[OD2]
	B: Arg 98[NH1]	2.63	E: Glu 96[OE1]
	B: Arg 98[NH2]	3.15	E: Glu 96[OE2]
2EWC	A: Asp 72 [OD2]	2.89	K: Gly 106 [N]
	A: Glu 100 [OE2]	2.89	K: Gln 98 [NE2]
	A: Gly 106 [N]	2.72	K: Asp 72 [OD2]
	A: Gln 98 [NE2]	2.66	K: Glu 100 [OE2]

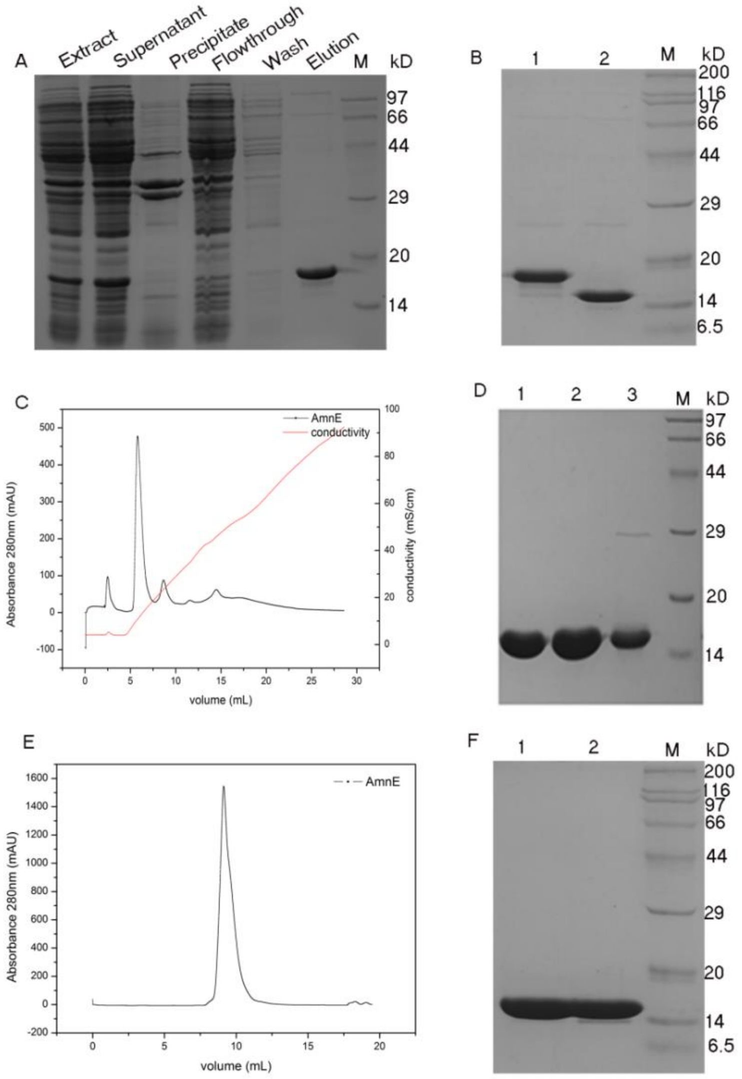

Supplementary figure 1. **Purification of wild-type AmnE.** (A) SDS–PAGE of affinity chromatography samples of wild-type AmnE. In this (and subsequent subfigures) M denotes a protein marker. (B) 6×His tag fusion AmnE cleavage of the tag by the enzyme Thrombin. Lanes 1 and 2 respectively depict the outcomes before and after enzymatic cleavage with Thrombin. (C) Anionic exchange chromatography peak map of wild-type AmnE. (D) SDS–PAGE of anion exchange chromatography samples of wild-type AmnE. Lanes 1, 2, and 3, depict outcomes from three different samples of the AmnE anion exchange chromatography. (E) Gel exclusion chromatography of peak map wild-type AmnE. (F) SDS–PAGE of gel exclusion chromatography samples of wild-type AmnE. Lanes 1 and 2 correspond to two different samples of the AmnE gel exclusion chromatography.

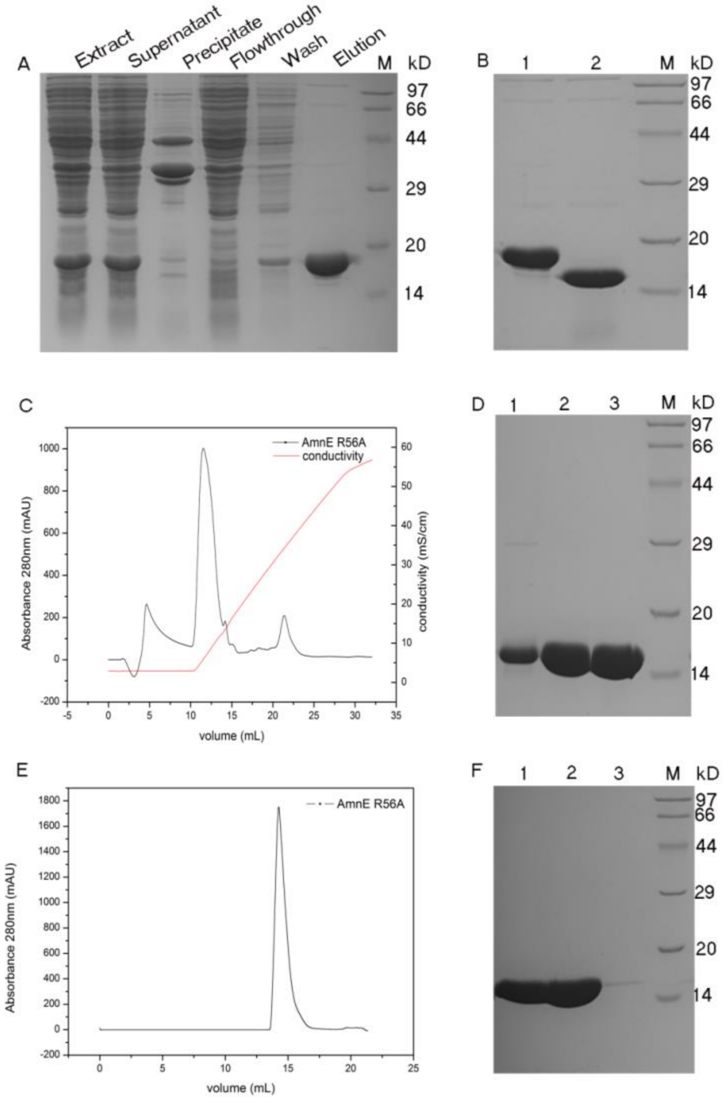

Supplementary figure 2. **Purification of mutant AmnE R56A.** (A) SDS–PAGE of affinity chromatography samples of mutant AmnE R56A. In this and subsequent subfigures, M denotes a protein marker. (B) 6×His tag fusion mutant AmnE R56A cleavage of the tag using enzymatic cleavage using Thrombin. Lanes 1 and 2 respectively depict the outcomes before and after enzymatic cleavage with Thrombin. (C) Anion exchange chromatography peak map of mutant AmnE R56A. (D) SDS–PAGE of anion exchange chromatography samples of mutant AmnE R56A. Lanes 1, 2, 3, respectively depict outcomes from samples from the AmnE anion exchange chromatography. (E) Gel exclusion chromatography of peak map mutant AmnE R56A. (F) SDS–PAGE of gel exclusion chromatography samples of mutant AmnE R56A. Lanes 1, 2, 3, depict outcomes from samples from the AmnE based on the use of gel exclusion chromatography.

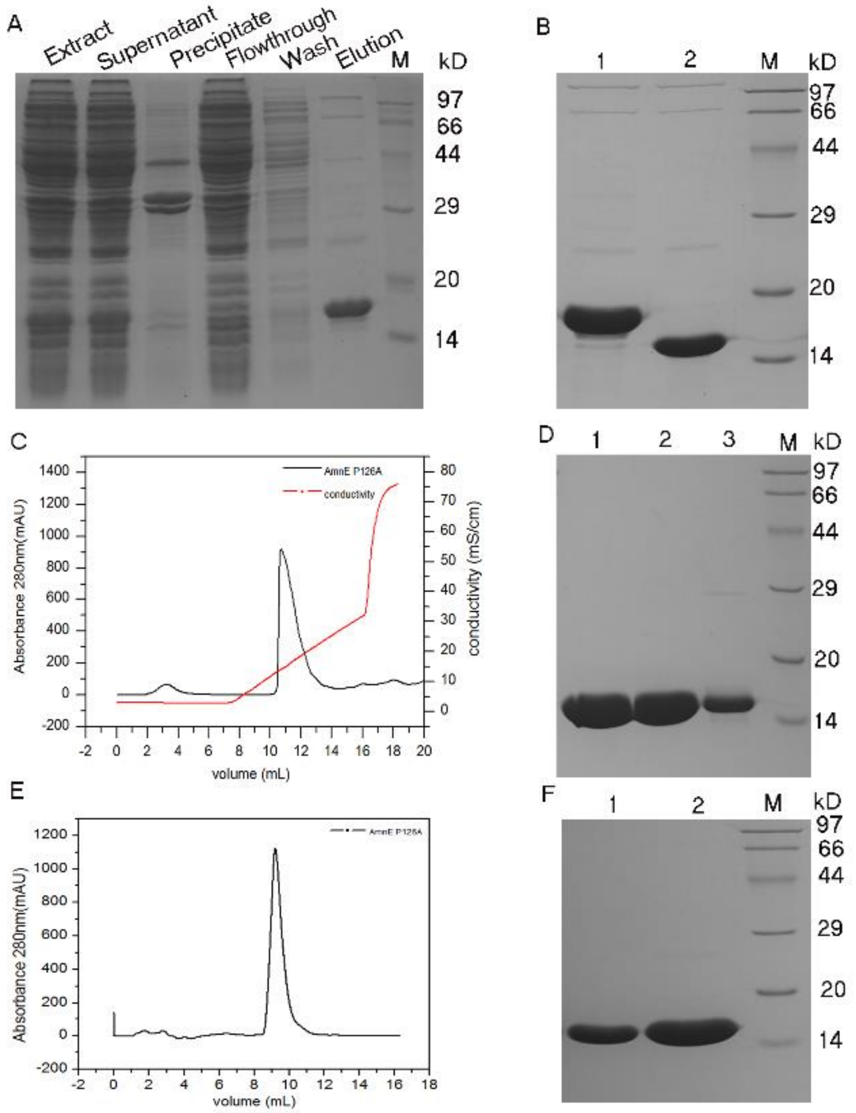

Supplementary figure 3. **Purification of mutant AmnE P126A.** (A) SDS–PAGE of affinity chromatography samples of mutant AmnE P126A. In this and subsequent subfigures, M denotes a protein marker. (B) 6×His tag fusion mutant AmnE P126A cleavage of the tag using enzymatic cleavage with Thrombin. Lanes 1 and 2 respectively depict the outcomes before and after the enzymatic cleavage with Thrombin. (C) Anion exchange chromatography peak map of mutant AmnE P126A. (D) SDS–PAGE of anion exchange chromatography samples of mutant AmnE P126A. Lanes 1, 2, and 3, respectively depict samples from the AmnE P126A anion exchange chromatography. (E) Gel exclusion chromatography of peak map mutant AmnE P126A. (F) SDS–PAGE of gel exclusion chromatography samples of mutant AmnE P126A. Lanes 1 and 2 depict samples of the AmnE P126A based on the use of gel exclusion chromatography.

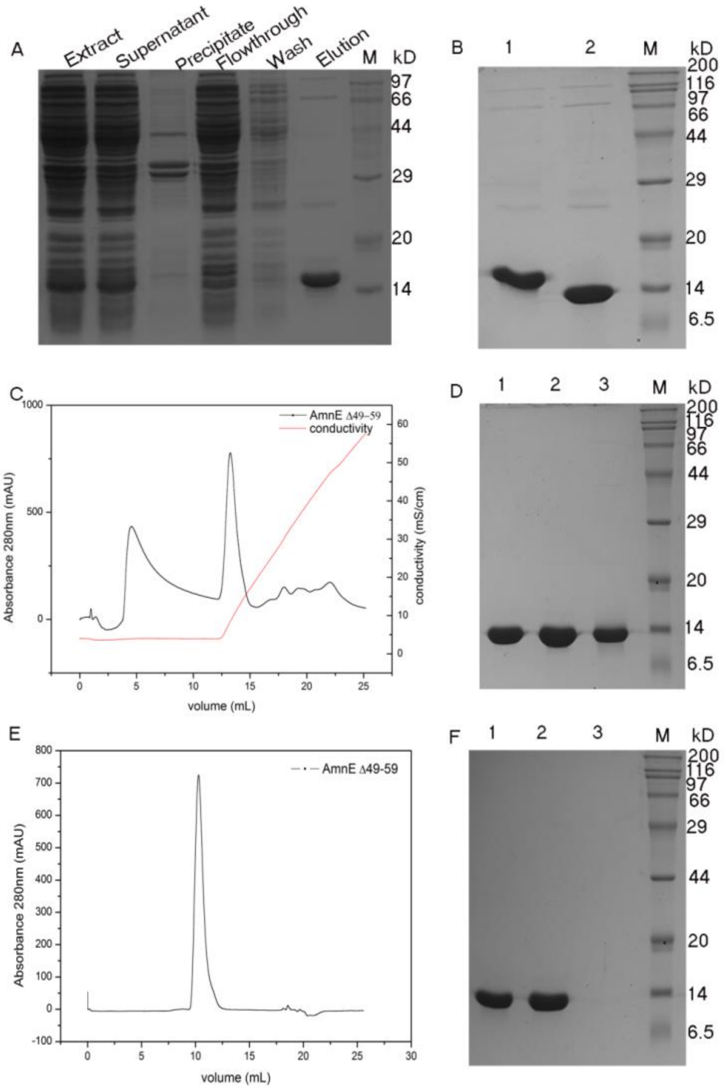

Supplementary figure 4. Purification of mutant AmnEΔ49–59. (A) SDS–PAGE of affinity chromatography samples of mutant AmnEΔ49–59. In this and subsequent subfigures, M denotes a protein marker. (B) 6×His tag fusion mutant AmnEΔ49–59 cleavage of the tag by the enzyme Thrombin. Lanes 1 and 2 respectively depict the outcomes before the enzymatic cleavage by thrombin. (C) Anion exchange chromatography peak map of mutant AmnEΔ49–59. (D) SDS–PAGE of anion exchange chromatographic samples of mutant AmnEΔ49–59. Lanes 1, 2, and 3, show the outcomes from different samples from the AmnE based on the use of anion exchange chromatography. (E) Gel exclusion chromatography of peak map mutant AmnEΔ49–59. (F) SDS–PAGE of gel exclusion chromatography samples of mutant AmnEΔ49–59. Lanes 1, 2, 3, respectively depict the outcomes of different samples of the AmnEΔ49–59 based on the use of gel exclusion chromatography.

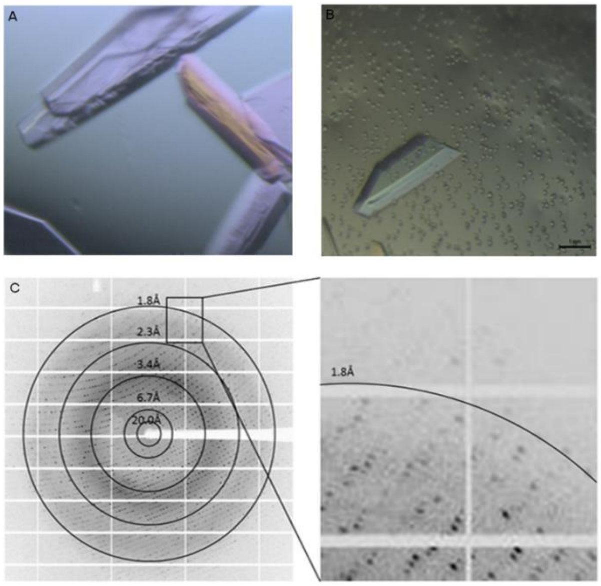

Supplementary figure 5. Crystallization and X-ray data collection of AmnE. (A) Initial crystal of AmnE. (B) Optimized single crystal of AmnE. (C) X-ray diffraction point atlas of the AmnE crystal (resolution, 1.75 Å).

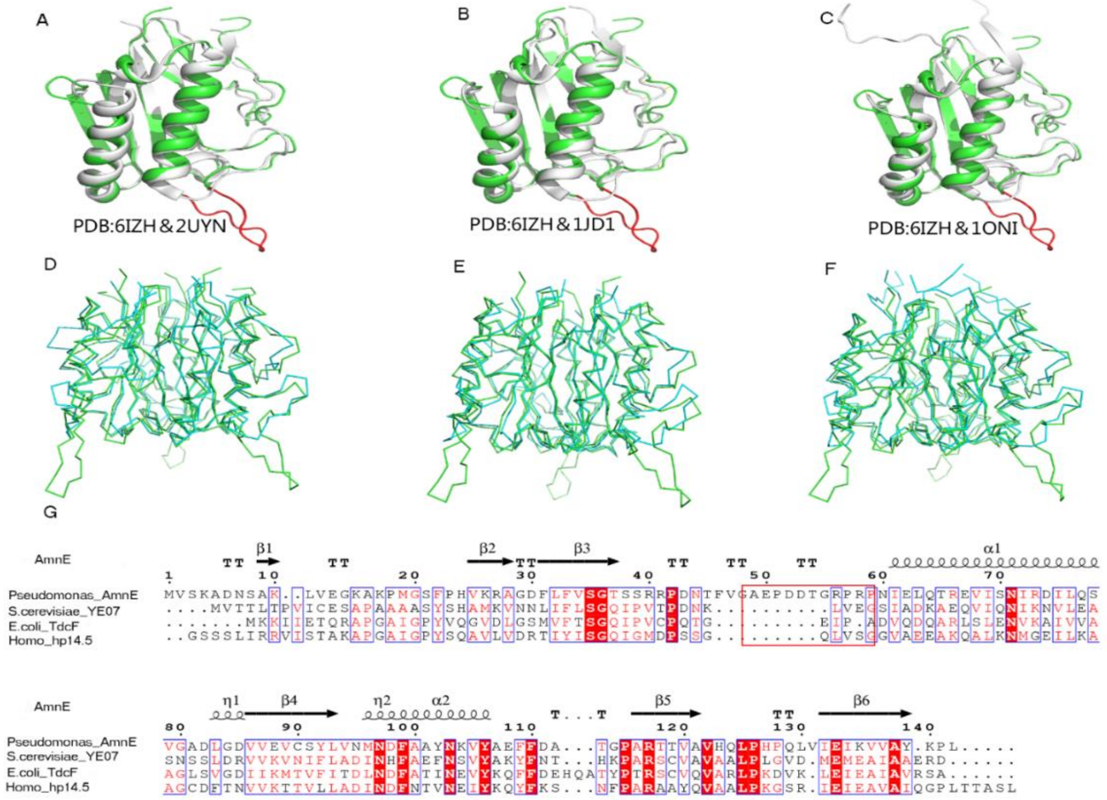

Supplementary figure 6. Structural superimposition and multiple sequence alignment. (A–C) Superposition of AmnE protomer from *Pseudomonas sp. AP–3* (green) with the protomeric structures of TdcF from *E. coli* (RMSD = 0.69 Å), YE07 from *Saccharomyces cerevisiae* (RMSD = 0.69 Å), and hp14.5 from human (RMSD = 0.85 Å). The major difference is the loop Lp2 of AmnE, which is drawn in red. (D–F) Superposition of the AmnE trimer (green) with the trimeric structures of TdcF (RMSD = 0.77Å), YE07 (RMSD = 0.69 Å), and hp14.5 (RMSD = 0.90Å). (G) Comparison of AmnE with other members of the YjgF family. Sequence alignment of AmnE with TdcF, YE07, and hp14.5. Residues 49–59 (red frame) of AmnE are in the loop Lp2, which is a unique region in AmnE. Sequences were aligned using the tool at the web site (https://www.ebi.ac.uk/Tools/msa/clustalo/) and the alignment was presented using the online ESPript 3.0 server http://espript.ibcp.fr/ESPript/ESPript/).

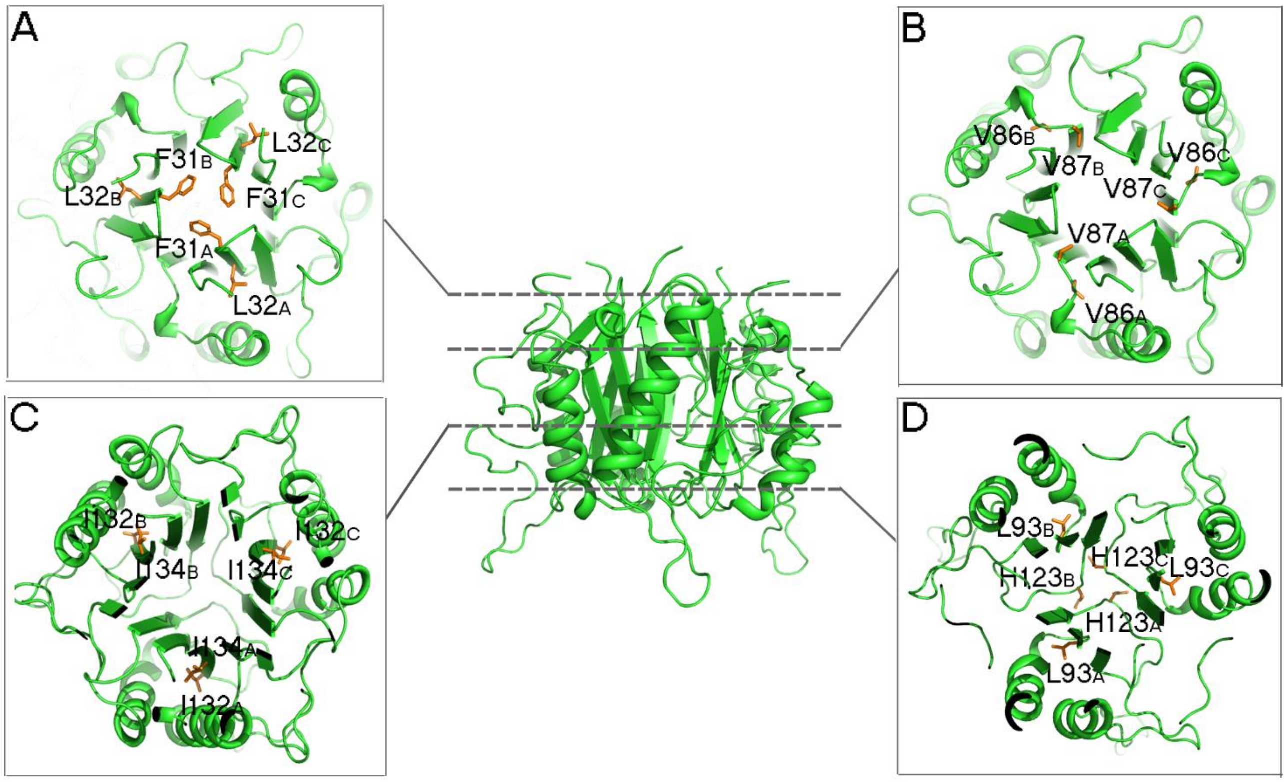

Supplementary figure 7. **AmnE trimer.** The inner hydrophobic residues located on the β-sheet work together to form hydrophobic barrel center. (A–D, from top to bottom) Four transverse sections show the details of hydrophobic barrel. The hydrophobic residues Phe^{31}, Leu^{32}, Val^{86}, Val^{87}, Leu^{93}, Ile^{132}, and Ile^{134}, are shown in orange sticks. The figure was produced with PyMOL (http://www.pymol.org).

A

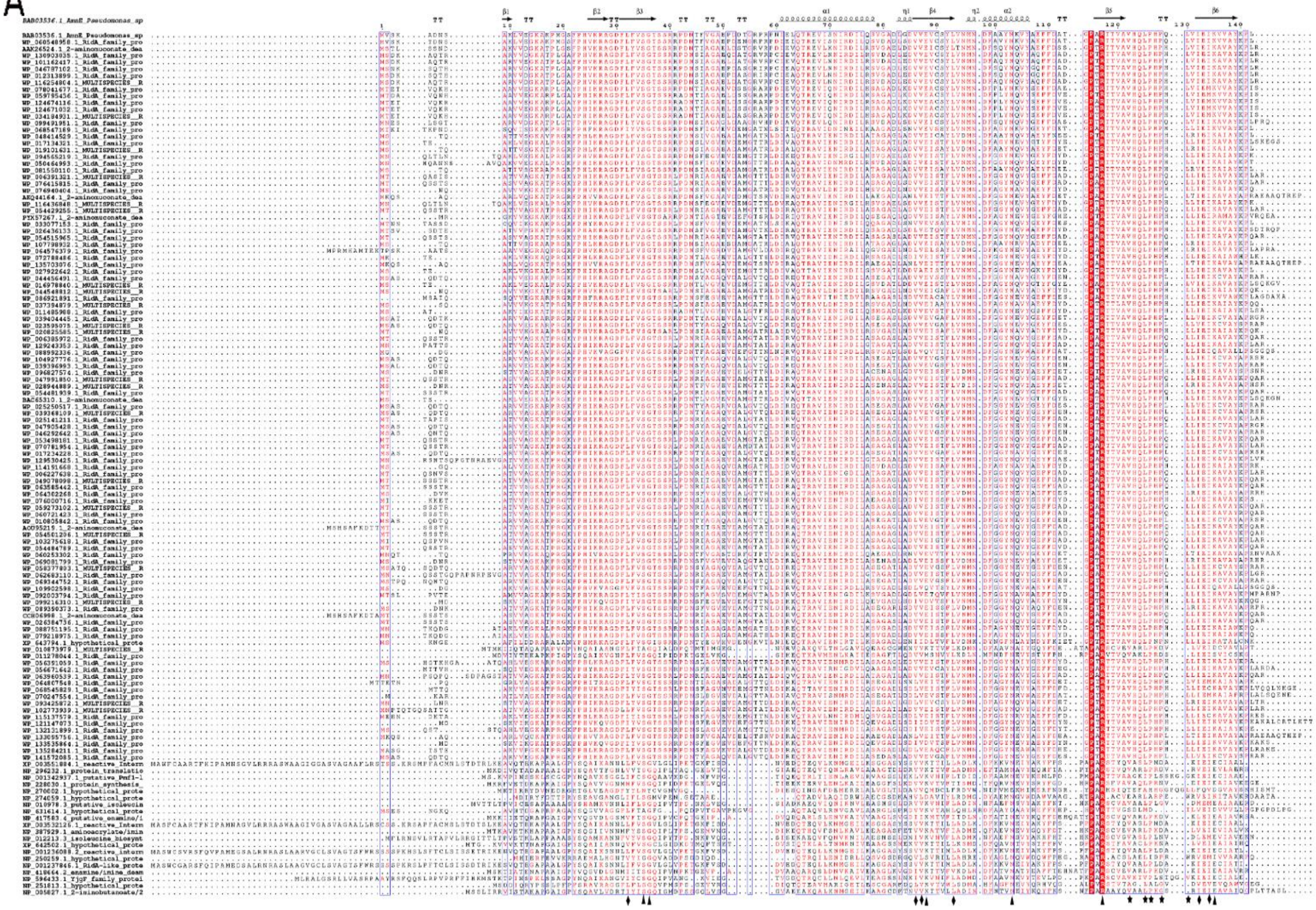

Supplementary figure 8. Multiple sequence alignment. (A) Multiple sequence alignment of the AmnE protein with RidA subfamily proteins. Residues labeled with rhombi are conserved hydrophobic residues on the inner trimeric surface of the barrel. Residues labeled with triangles are highly conserved in clefts located at the subunit interfaces of the AmnE trimer. Residues labeled with five-pointed star are conserved residues formed a hydrophobic base on AmnE loop Lp3.Residues Sequences were aligned using the tool obtained from the website (https://www.ebi.ac.uk/Tools/msa/clustalo/) and the alignment was presented using the online ESPript 3.0 server (http://espript.ibcp.fr/ESPript/ESPript/)

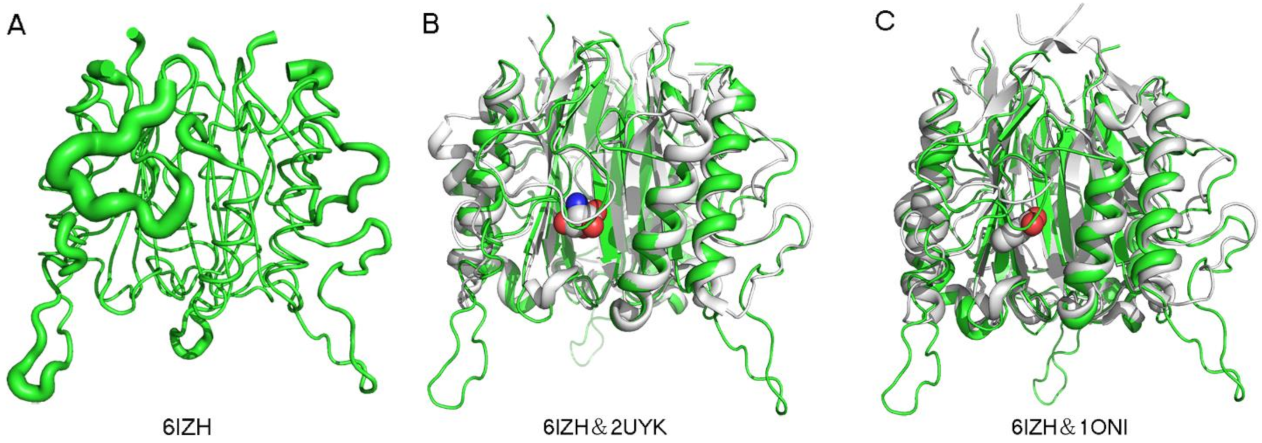

Supplementary figure 9. Ligand binding site of AmnE. (A) AmnE trimeric structure is rendered as a putty according to the R-factor. Residues with the highest B-factors in the AmnE trimer are mostly located in the Lp1 loop region, which is predicated as a part of the ligand binding pocket. (B) Superposition of the AmnE trimer with the structures of the TdcF complex with 2-ketobutyrate (RMSD = 0.77 Å).AmnE colored in green ,TdcF colored in gray and 2-ketobutyrate shown in sphere. (C) Superposition of the AmnE trimer with the structures of the hp14.5 complex with benzoate (RMSD = 0.90Å). AmnE colored in green, hp14.5 colored in gray and benzoate shown in sphere. All figures were produced with PyMOL (http://www.pymol.org).
